# Effects of Distance-Learning Strategies in Dental Fixed Prosthodontics Amidst the COVID-19 Pandemic: Cross-Sectional Questionnaire Study on Preclinical Dental Students’ Perspective

**DOI:** 10.2196/45311

**Published:** 2023-11-08

**Authors:** Xi Yue Zhang, Anelyse Arata Found, Sheila Butler

**Affiliations:** 1 Schulich School of Medicine and Dentistry Western University London, ON Canada

**Keywords:** dental education, dental, dentist, dentistry, technology-based learning, online learning, pre-clinical training, distance learning, transmissibility, dental school, mental health, COVID-19, student perception, online teaching, survey, teaching methods, training, isolation, teaching, module, education

## Abstract

**Background:**

COVID-19’s high transmissibility led to gathering restrictions where dental schools experienced disruptions due to restrictions on attending in-person lectures and limitations placed on applied preclinical and clinical activities. Students not only had to rapidly switch to digital technology-based learning (TB-learning) modules but also experienced high levels of social isolation and anxiety around virus transmission.

**Objective:**

This study aims to evaluate the preclinical students’ perception of switching TB-learning modules amidst the COVID-19 pandemic, identifying which module parameters were associated with strong student outcomes.

**Methods:**

A web-based survey of 39 Likert scale questions was delivered to preclinical dental students (Western University) to evaluate students’ perceptions concerning TB-learning, fear amidst the COVID-19 pandemic, and the impact on their preclinical training. A Spearman rank correlation coefficient was determined to estimate the relationship between 2 variables in isolation (*P*=.01). An ordinal regression analysis was performed on variables of interest to determine how module variables (typically within the instructor’s control) influenced the student outcomes (*P*=.05).

**Results:**

The response rate was 30% (n=39). TB-learning was considered vital (34/39, 87.2%) as the students’ education improved (18/39, 46.2%). However, 53.8% (n=21) of students showed increased difficulties in retaining, visualizing, or understanding the materials using TB-learning, and 64.1% (n=25) found it more difficult to concentrate than in in-person classes. In total, 79.5% (n=31) of students showed different levels of agreement about feeling fatigued from TB-learning. Through Spearman ρ correlation analysis, the quality of questions in quizzes (ρ=0.514; *P*<.001), relevant handouts (ρ=0.729; *P*<.001), and high-quality audiovisuals (ρ=0.585; *P*<.001) were positively correlated with students responding that the modules were useful to preclinical training. Similarly, good organization (ρ=0.512; *P*<.001), high-quality questions in quizzes (ρ=0.431; *P*=.01), and relevant handouts (ρ=0.551; *P*<.001) were positively correlated with web-based classes as an effective way to learn. In total, 91.6% (n=36) of the students agreed that COVID-19 was a dangerous disease, whereas 53.8% (n=21) showed different levels of agreement that they were afraid to be infected personally, and 69.2% (n=27) feared passing COVID-19 along to family and friends. A total of 82.1% (n=32) of the students showed that COVID-19 impacted their overall learning process and had a negative impact on their practical preclinical training (31/39, 79.5%).

**Conclusions:**

The students found a difference between TB-learning and face-to-face learning methods, where the students perceived fatigue toward the web-based method with difficulty concentrating and visualizing the subject. Moreover, there was a consensus that COVID-19 itself affected the students’ overall learning process and preclinical training. As dental schools continue implementing TB-learning into their curriculum, this investigation identifies the students’ struggles with the paradigm shift. In an effort to improve TB-learning, this work highlights 4 variables (organization, quizzes, quality handouts, and quality audiovisuals) within the control of instructors that can help improve content deliverance, improving the students’ experience.

## Introduction

The year 2020 was marked by different responses regarding COVID-19 causing SARS-CoV-2. In early March, the World Health Organization (WHO) declared COVID-19 a pandemic, and implementing social distancing was the most crucial strategy to prevent the spread of the virus [1,2]. The dental schools and professors had to rapidly transform in-person lectures into web-based didactic education to help flatten the transmission curve [3-8]. As a result, most classes were moved to web-based platforms [9-11].

Successful distance learning relies heavily on the faculty members’ ability to express the content, the education deliverance design, and the students’ experience with the technology [12,13]. However, the anxiety due to the outbreak, fear of transmitting the virus, high stress levels, and increased isolation decreased the students’ motivation and academic results [3,14-21]. Despite the hiccups related to the technology-based learning (TB-learning) transition, the dental education field has developed a new suite of web-based resources and forced the enhancement of individual technological skills [3]. Although students adapted to the new TB-learning faster than the senior professors since they were more comfortable with the technology, this gap may be reduced after the pandemic as web-based lectures and demonstrations become an acceptable tool for dental education [22]. Future resources might be improved due to faculty and students having already overcome the steep initial learning curve as well as a reduced time crunch in module development [3,23].

Nevertheless, web-based learning cannot solely replace preclinical and clinical training that allows students to learn and maximize their motor skills, with a need to understand how TB-learning influenced dental students’ learning during the COVID-19 pandemic. After the pandemic, the continued use of digital learning led to the necessity of understanding the students’ perspective to improve the education deliverance methodology in dental education. Therefore, this study aims to evaluate the preclinical students’ perception concerning the TB-learning modules and fear amidst the COVID-19 pandemic, identifying which module parameters were associated with strong student outcomes.

## Methods

### Survey Population and Format

The open survey was sent to 132 students of the fixed prosthodontics preclinical laboratory classes at Western University, Schulich School of Medicine & Dentistry, London, Ontario, Canada. The questionnaire was sent by email to each potential participant using the Qualtrics platform to evaluate students’ perceptions concerning TB-learning, fear amidst the COVID-19 pandemic, and the impact on their preclinical training. The survey was rolled out in June and July 2021, in a total of 6 weeks.

The TB-learning courses evaluated comprised weekly web-based didactic modules broken into parts with slides and video recordings. Each web-based module comprised the theoretical part and a detailed visual description of the procedures and techniques that the students would be using in the preclinical laboratory. The slide handouts were given beforehand and could be fully annotated. The lectures were related to fixed prosthodontics didactic. The students were required to come into the simulation laboratory each week, adding a total time of 40-50 hours of hands-on preclinical training. Learning was reinforced with 5 interspersed quizzes and several knowledgeable questions after each recorded lecture. Due to the social distancing mandate, the number of students and instructors was reduced per laboratory section.

The open survey included a participant information sheet, an informed consent form, a data protection sheet, and a questionnaire of 39 questions divided into 5 sections (Table 1; Multimedia Appendix 1 [14,15,24-27]). The first section gathered the participants’ demographic data, and the second evaluated their technology literacy regarding computer skills, access to the internet, access to electronic devices, and experience level in TB-learning. In the third section, students were asked their opinions on specific aspects and features of the web-based resources. The following section asked students to evaluate their perception of their overall learning process in the course and preclinical training. Finally, students were asked questions regarding their perception of COVID-19. The answers were presented on a Likert scale with a neutral central value and were considered ordinal data.

**Table 1:** Survey question themes and response breakdown.

Question theme	Response options
Demographic details	Text-based/options
Technology literacy background	5-point Likert scale
Regarding aspects of the web-based resource and overall learning process	7-point Likert scale
Regarding their preclinical training	7-point Likert scale
Regarding COVID-19	7-point Likert scale

### Statistical Analysis

The ordinal data were analyzed using descriptive statistical analysis and interferential correlation through the Spearman ρ coefficient to establish the relationship between responses and allow an estimated correlation between 2 variables in isolation. The correlations were limited to those that were statistically significant (*P*=.01) and were more than moderately correlated (ρ>0.4) [28] (SPSS statistics software, version 28). A more comprehensive ordinal regression analysis was performed to identify how certain module variables (typically within the instructors’ control) influence the student outcomes (*P*=.05). The 2 student outcomes variables, which were questions that most directly asked how the student perceived their web-based learning and education, were selected for the analysis:

- Web-based classes were an effective way to learn about the assigned topics.
- The content of the web-based classes was very useful for my preclinical training.

### Ethical Considerations

The web-based survey is in accordance with the Cherries checklist and was approved by The Western University Health Science Ethics Board (HSREB) in Canada (#118126). The purpose of the study was outlined in both the email and the survey introduction. Informed consent was obtained from all participants upon final survey submission. Participants could return to previous questions or review their answers at any point. Study data and identifiers were anonymized during the data collection and data analysis. No compensation was awarded to participants. Data were stored on secure Canadian servers that were password-protected.

## Results

**Figure 1:**
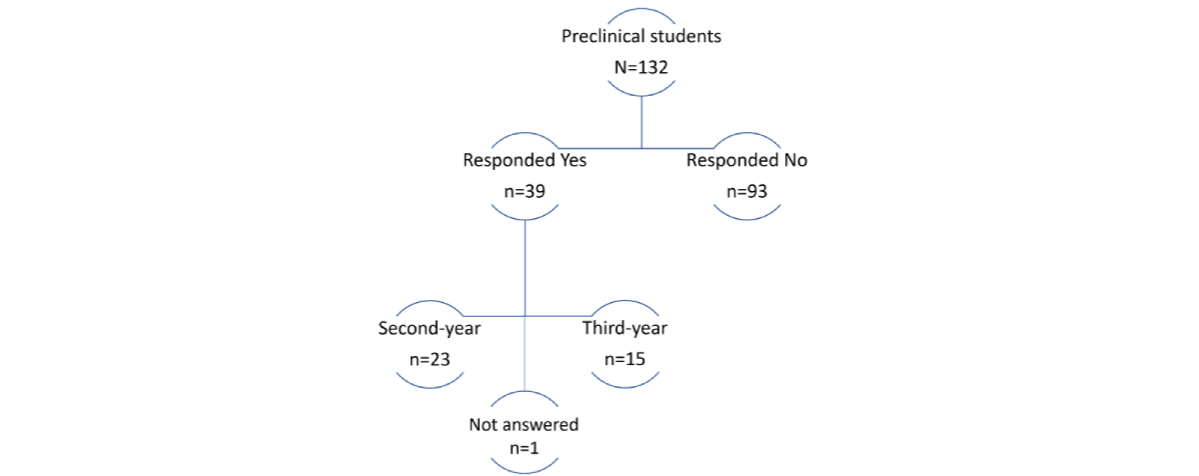
Survey responses from second- and third-year preclinical dental students, where the response “No” was for the participants who responded no to the study.

Among the 132 students contacted, 39 individuals (26/39, 66.7% females and 13/39, 33.3% males) consented and responded to the survey (response rate of 30%). Of the students who consented to participate in the study, 59% (n=23) were in their second year of the dentistry program, and 38.5% (n=15) were in the third year, with 1 person who did not specify their academic year (Figure 1).

**Figure 2:**
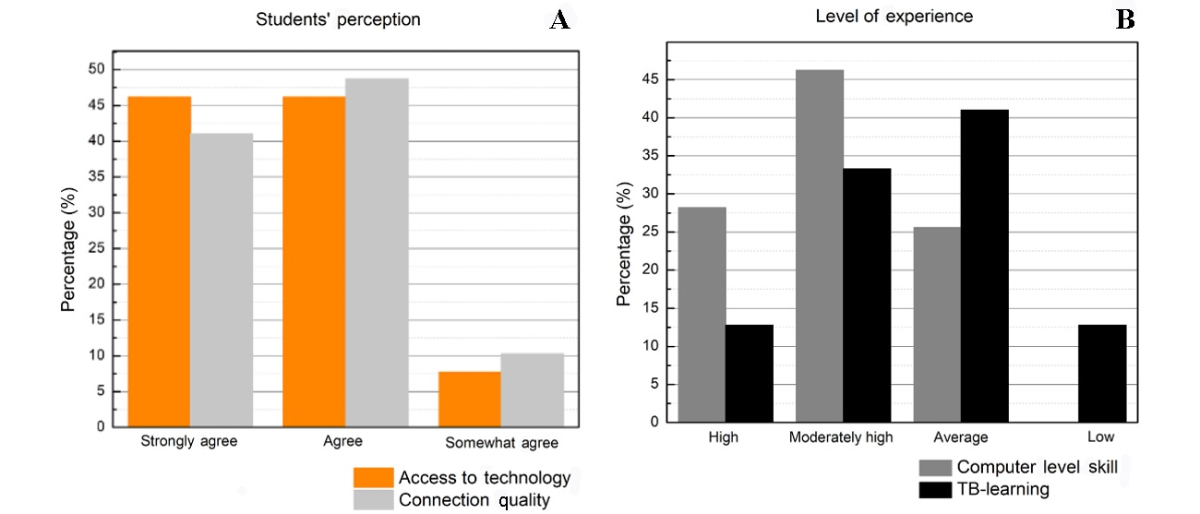
Graphics show the students’ perception concerning their (A) access to technology and access to good internet connection quality; and (B) students’ level of experience in their computer level skill and TB-learning. TB-learning: technology-based learning.

The students reported having access to technology and a good internet connection (Figure 2A). Self-reported computer skill level was high, with 74.4% (n=29) of students stating that they had a moderately high to high level of experience and 25.6% (n=10) having an average level of experience (Figure 2B).

**Figure 3:**
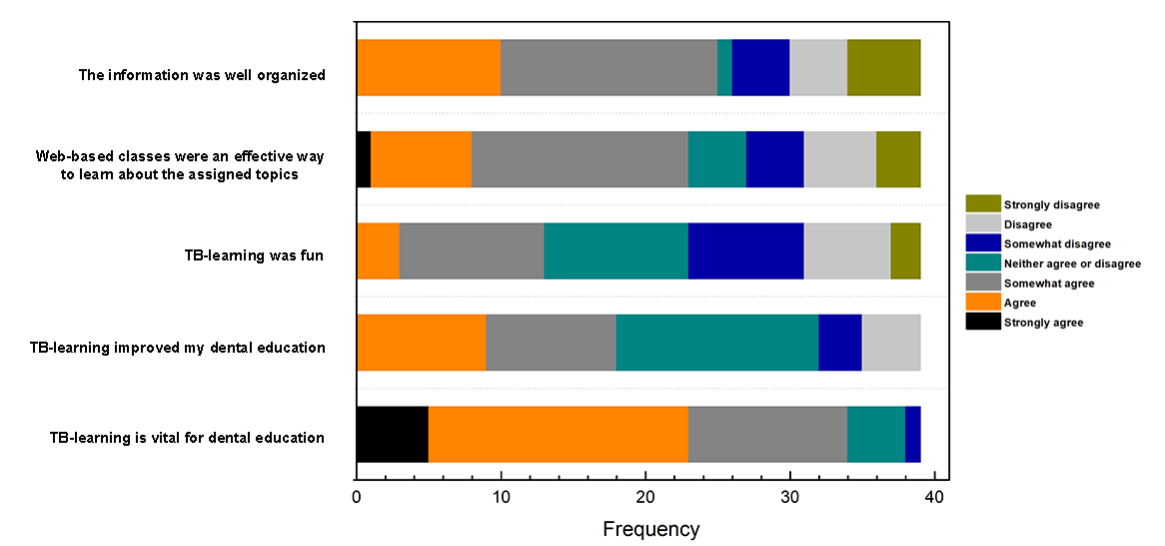
The graphic shows the students’ perception concerning their web-based classes and TB-learning experience. TB-learning: technology-based learning.

In Figure 3, the students responded positively to the web-based learning resource, where students showed different levels of agreement that TB-learning was vital (34/39, 87.2%) and improved (18/39, 46.2%) their dental education. However, there was a polarized perception of web-based classes as an effective way to learn the assigned topics. Despite the difference of opinion, there were different levels of agreement that the structure of the information delivery was well organized.

**Figure 4:**
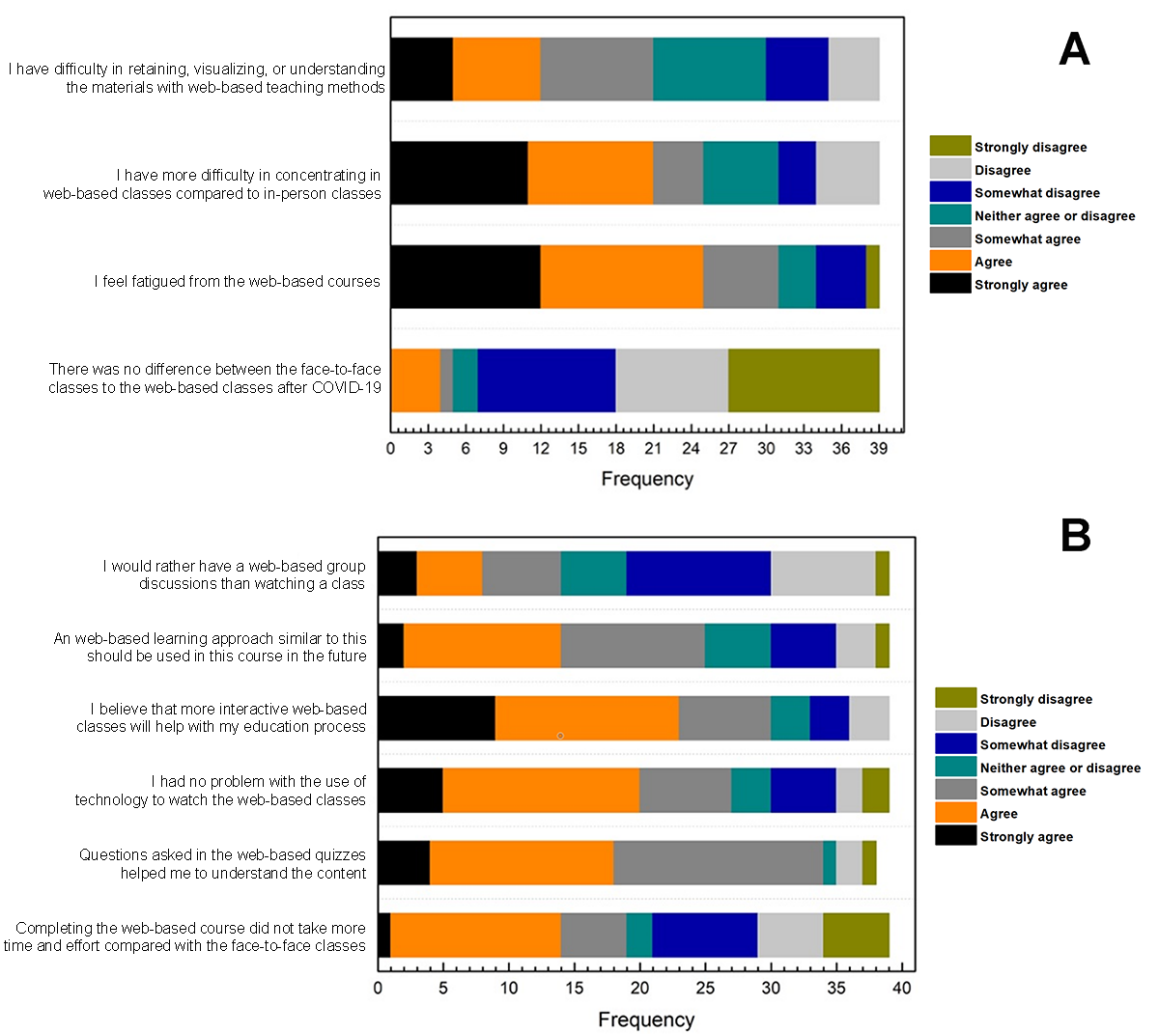
The graphic shows the students’ perception concerning (A) their engagement with web-based classes; and (B) the modules’ material and preference toward their education process.

Figure 4A shows that 53.8% (n=21) of the students presented different levels of agreement concerning their difficulties in retaining, visualizing, or understanding the materials with the web-based teaching methods. In total, 64.1% (n=25) perceived that web-based classes were more challenging to concentrate on compared to in-person classes and 30.8% (n=12) of the students strongly agreed with feeling fatigued due to web-based teaching. In addition, a high number of students (12/39, 30.8%) strongly disagreed that there was no difference between face-to-face and web-based classes during COVID-19. A total of 33.3% (n=13) of students showed a neutral position regarding the level of engagement in their education during the pandemic. On the other hand, 51.3% (n=20) of the students showed that their engagement level was somewhat to extremely worsened.

Students agreed that quizzes helped them to understand the content (34/39, 87.2%; Figure 4B). In addition, they also agreed (30/39, 76.9%) that more interactivity in their classes would have helped with their education. Despite that, when asked about having web-based group discussions instead of watching a class, most students (20/39, 51.3%) disagreed with that sentiment, and 35.9% (n=14) showed different levels of agreement. Polarized perceptions concerning the time and effort taken for web-based classes compared with the in-person model were identified, where 46.2% (n=18) of students found the web-based course was more time-consuming than the in-person classes. Most students (27/39, 69.2%) stated they did not have problems accessing the content web-based. In contrast, 1-quarter of the interviewed students encountered problems using technology to watch the web-based lecture.

**Figure 5:**
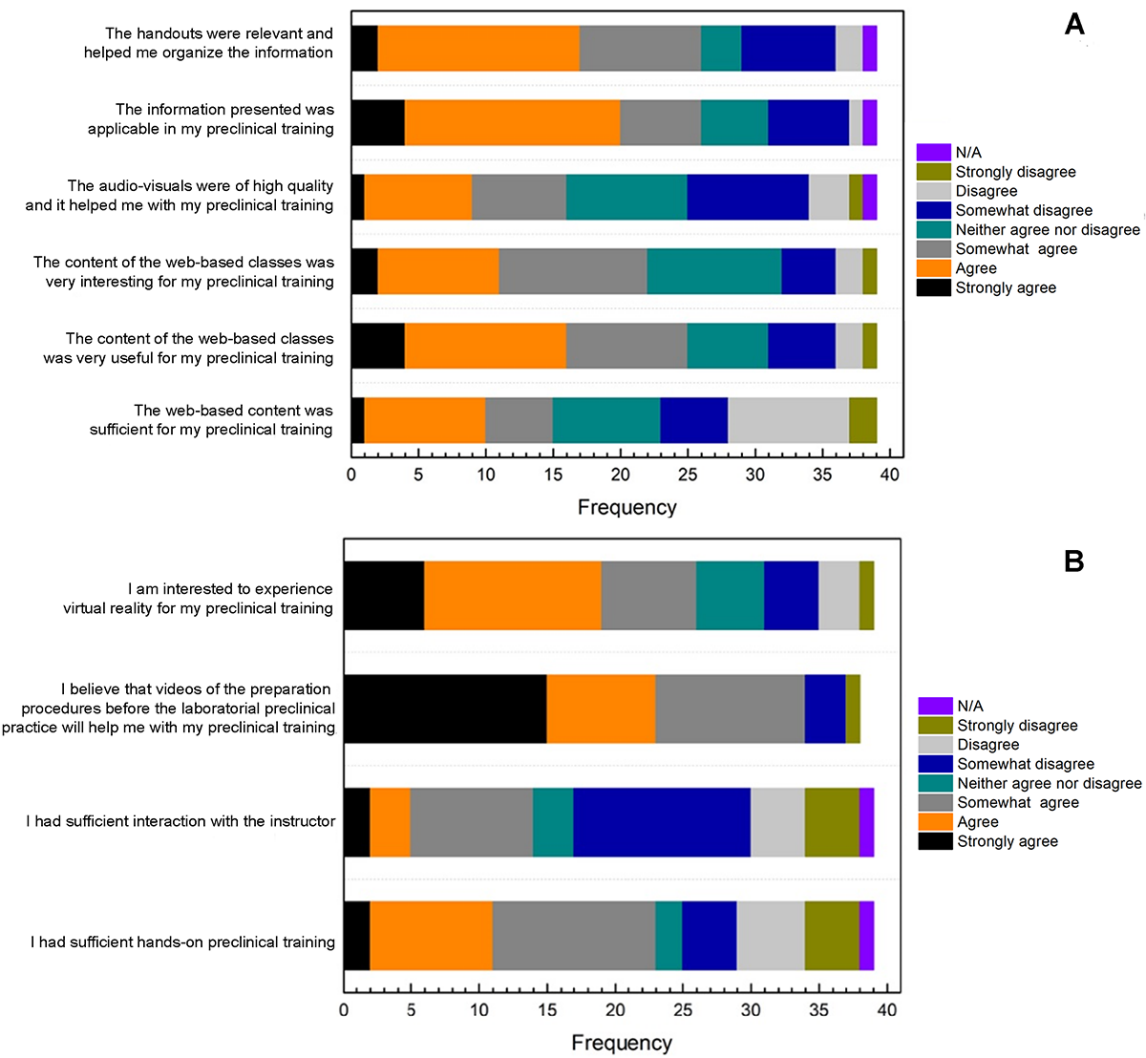
The graphic shows the students’ perception of (A) the web-based classes regarding their preclinical training; and (B) their hands-on preclinical training and suggestions for improvement.

Regarding the preclinical training, most students showed different levels of agreement that the content of the web-based classes was useful (25/39, 64.2%) and interesting (22/39, 56.4%; Figure 5A). The audiovisuals were of high quality and helped 41% (n=16) of the students. Furthermore, 66.7% (n=15) of the students agreed that using handouts was relevant and helped them organize the information.

Figure 5B shows that 59% (n=23) of the students demonstrated different levels of agreement that they had sufficient hands-on preclinical training. The students strongly agreed that the use of videos showing the preclinical procedures helps with their preclinical training (15/39, 38.5%), and 66.6% (n=26) indicated an interest in experiencing the use of virtual reality in their preclinical training.

Spearman ρ coefficient analysis showed that age, computer skill, and TB experience were not significantly correlated with any questions regarding their experience with the web-based module (ie, ρ<0.4 and *P*>.01). The greater the age of the students, the lower the score with the following statements “COVID-19 negatively affected my overall learning process in dentistry.” (ρ=–0.440; *P*=.005) and “COVID-19 negatively affected my preclinical learning.” (ρ=–0.421; *P*=.008) where the lowest scores represented the students disagreeing with the statements. Older students found that they were less impacted by COVID-19. Significant correlations were not observed directly between COVID-19 fears and other student outcome variables. In addition, a positive correlation was observed in students who agreed to the following, “I feel fatigued from the online courses” had a greater response to “I have difficulty in retaining, visualizing, or understanding the materials with the online teaching methods” (ρ=0.609; *P*<.001).

The module variables that impacted the selected student outcome variables are presented in Table 2. The results suggested that out of all module variables, the organization of the modules, quizzes, handouts, and audiovisual quality was most correlated to the student’s positive response concerning the web-based classes being effective and useful.

An ordinal regression analysis with the 3 module variables and their impact on 2 student outcomes was performed (Table 3), showing that organization impacted web-based learning and increased the student perception of learning effectiveness. A minimal impact was observed concerning the organization of the lecture and on the students’ perception of the usefulness of the material in their preclinical training. Quizzes were perceived as an effective way to learn in web-based classes and a useful source for preclinical training. Last, audiovisuals of high quality led to increased student reports of module usefulness in their preclinical training. Nevertheless, this statement did not impact the students’ opinion on TB-learning effectiveness. In both analyses, quizzes were the module variable with the most significant odds ratio, leading to a likely larger impact on results. The module variables exhibited a more significant impact on usefulness than effectiveness.

**Figure 6:**
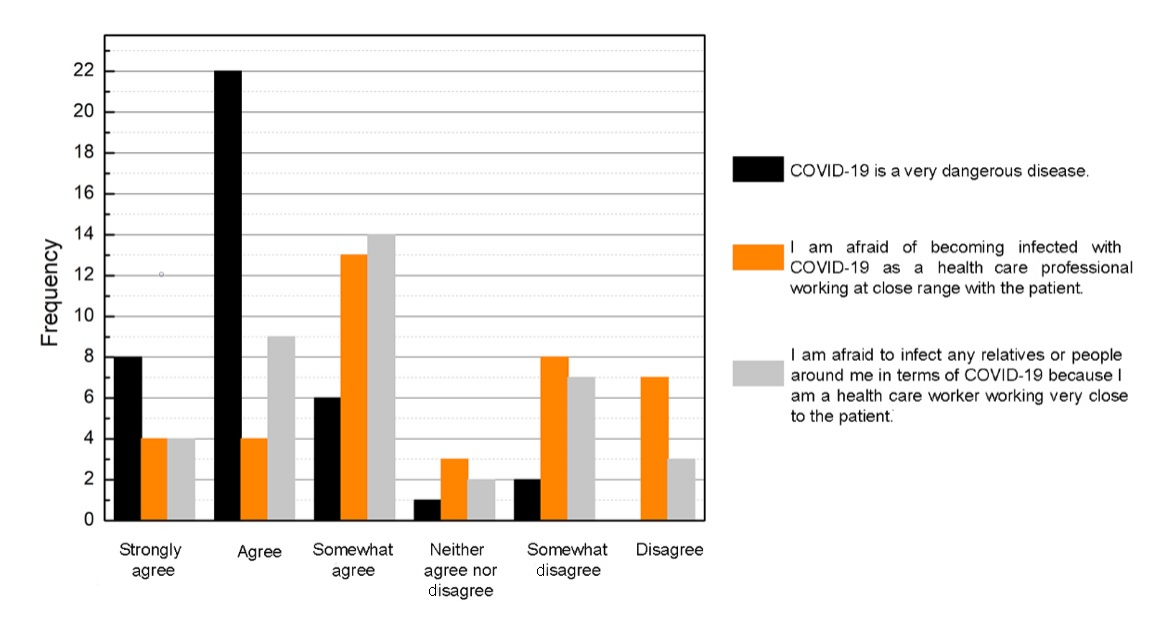
Graphic showing the students’ perception concerning COVID-19.

Figure 6 exhibits that 92.6% (n=36) of the students agreed that COVID-19 was a dangerous disease. When asked about specific fears regarding the risk of COVID-19 infection as a health care professional, 53.8% (n=21) of students showed different levels of agreement that they were afraid to be infected personally, and 69.2% (n=27) were afraid to pass COVID-19 along to those around them (family and friends). In total, 82.1% (n=32) of the students answered that COVID-19 impacted their overall learning process. The same responses were observed in the students’ preclinical training; 79.5% (n=31) of the students perceived that COVID-19 had a negative impact on their education.

**Table 2:** Spearman ρ correlation table summary.

Module variable			ρ		*P* value
**Web-based classes were an effective way to learn about the assigned topics**					
	The information was well-organized	0.512		<.001	
	Questions asked in web-based quizzes helped me understand the content	0.431		.013	
	The handouts were relevant and helped me organize the information	0.551		<.001	
**The content of the web-based classes was very useful for my preclinical training**					
	Questions asked in the web-based quizzes helped me understand the content	0.514		<.001	
	The handouts were relevant and helped me organize the information	0.729		<.001	
	The audiovisuals were of high quality and helped me with my preclinical training	0.585		<.001	

**Table 3:** Odds ratio table of ordinal regression analysis.

Participant outcome	Module variables					
	Well organized module	*P* value	High-quality quizzes	*P* value	High-quality audiovisuals	*P* value
The web-based module was effective	OR^a^ 1.74 (95% CI 1.17-2.67)	.008	OR 1.92 (95% CI 1.15-3.38)	.016	Minimal/no significant impact	N/A^b^
The web-based module was useful	Minimal/no significant impact	N/A	OR 3.49 (95% CI 1.79-7.71)	<.001	OR 2.42 (95% CI 1.42-4.35)	.0017

^a^OR: odds ratio.

^b^N/A: not applicable.

## Discussion

### Principal Findings

This study demonstrated that the students’ self-reported experience regarding TB-learning varied, with 87.2% (n=34) reporting average to high-level experience. Age, computer skill, and TB experience were not significantly correlated with a positive experience in TB-learning, contradicting the findings of Al-Taweel et al [24] that showed that the higher the TB-learning experience and computer skills, the greater the students’ satisfaction, impacting the overall TB-learning evaluation. This discrepancy could be due to the homogenous nature of the dental student study group with good access to technology, internet connection, and strong computer skills.

Despite TB-learning being considered vital for the students’ learning process (34/39, 87.2%), only 38.5% (n=15) of students somewhat agreed that web-based classes were an effective way to learn the assigned topics. That could be attributed to the fact that TB-learning was not considered a fun experience for 41% (n=16) of the students. Although most students agreed that the web-based modules were well organized (25/39, 64%), they still found that their engagement decreased (20/39, 51.3%). In addition, 64.1% (n=25) of dental students reported difficulty concentrating in web-based classes compared with in-person classes.

The rapid change from in-person to web-based courses promoted a general feeling of fatigue in the students (31/39, 79.5%). The responses for “I feel fatigued from the online courses” and “I have difficulty in retaining, visualizing, or understanding the materials with the online teaching methods” exhibited a high significant correlation (ρ=0.609; *P*<.001). Although there was no directionality in the correlation, it could be attributed to the fact that the more fatigued students had more difficulty retaining or visualizing the materials, or that the increased difficulty resulted in more fatigue, or it could be due to an outside factor that increased both fatigue and difficulty. The findings suggested that similar questions regarding the overall learning process from students were scored similarly. This does highlight consistency among participants when answering the questionnaire. In contrast, this fact does not directly address the role the TB-learning resource played.

The literature describes that students declared increased exhaustion from TB-learning and worsening learning experience during the COVID-19 pandemic [25,29]. The lack of hands-on training, student interaction, problems with technology, and classroom time management were barriers to web-based dental education. Additionally, the lack of a structured web-based curriculum, less interaction with the professors, challenge to focus on schoolwork, and motivation to study influenced the students’ overall level of satisfaction during the COVID-19 pandemic, increasing the concerns toward the web-based courses [20,30].

The faculty also struggled to keep the students engaged and motivated since the students were more familiar with the interactive, synchronous learning experience [17,31]. The faculty had to adapt to the implementation of home offices, facing challenges such as developing web-based content in a short period. Furthermore, many faculty lacked experience and expertise in TB-learning and had to undergo a learning curve to use the available platforms [17,20,32]. However, the COVID-19 crisis might offer dental schools opportunities to leverage technology innovatively, helping enhance TB-learning in dental education [20]. In addition, “Generation Z” students can rapidly use technological developments, becoming the ideal candidates to maximize the effectiveness of remote education [33].

Interactive classrooms in web-based courses using clinical applications and team-based engagement components increased students’ performance during the pandemic [17]. Nonetheless, in this evaluation, when asked about web-based group discussion, most students either disagreed (20/39, 51.3%) or showed neutrality (5/39, 12.8%) that they would prefer this model instead of watching a class. Nijakowski et al [34] reported that dental students preferred remote learning as an asynchronous form (e-learning portals) to synchronous forms, such as real-time web-based meetings.

Despite that 47.1% (n=18) of the students found the web-based model more time-consuming, with 23% (n=9) encountering some problems accessing web-based content, the majority of the students showed that web-based classes (25/39, 64.1%) and content delivered (26/39, 66.7%) were useful and sufficient for their preclinical training during COVID-19. Audiovisuals and handouts were helpful for 41% (n=16) of the students in their preclinical training, where handouts helped the students (26/39, 66.7%) organize the information. Out of all the other module variables, the organization of the modules, quizzes, handouts, and audiovisual quality correlated most with the student’s positive response to effective and useful web-based classes.

The rapid transition to TB-learning promoted the flipped classroom teaching approach, which significantly improved the students’ learning experience compared to the traditional teaching methods [20,31,35]. Furthermore, e-learning techniques that engage students and instructors “beyond the classroom” should be considered, such as home-based simulation, videos of practical demonstrations, and procedural videos [13,20,36,37]. Moreover, computer-aided simulation techniques, humanoid robots that simulate a patient, visual reality, and the use of mobile platforms to simulate clinical procedures can be used as aids for the students’ preclinical and clinical training [10,38-40].

Unlike Spearman ρ, which compares 2 variables directly, the ordinal regression analysis models how multiple variables interact to determine an outcome. Though the module variable for “The handouts were relevant and helped me organize the information” seemed to impact the student’s responses to both the questions on effectiveness and usefulness in the Spearman ρ correlation analysis, when it was modeled, it no longer played a significant role in influencing either participant outcomes. Therefore, this question was not included in the analysis. The positive perceptions of organization and quizzes increased the students’ feeling of effective learning. In addition, the positive perceptions of using quizzes and audiovisuals in TB-learning increased the students’ reports of the module’s usefulness. In both analyses, quizzes were the module variable with the most significant odds ratio. In total, 2 general trends were found; first, the module variables exhibited a more significant impact on usefulness than effectiveness; second, of the module variables, quizzes had the largest impact on overall positivity. Additionally, dental students strongly agreed that videos showing preclinical procedures and virtual reality could improve their preclinical training.

Despite the modifications in the preclinical training triggered by the COVID-19 pandemic, 59% (n=23) of the students agreed that they had sufficient hands-on preclinical training. In contrast, 33.4% (n=13) of the students disagreed with the statement. In addition, 53.8% (n=21) of the students perceived insufficient interaction with the instructor at the preclinical practice. That might be a result of the restrictions imposed to control the spread of the disease that reduced the number of instructors per student. Nevertheless, the amount of interaction with the instructor also depends on the students’ engagement with the assigned preclinical project.

Most students perceived that the COVID-19 pandemic impacted their overall learning process (32/39, 82%) and negatively impacted their preclinical training (31/39, 79.5%). Despite the difficulties caused by the COVID-19 pandemic, most students (32/39, 82.1%) showed that they do not regret choosing dentistry as their future profession. There was consensus by the majority of the students (36/39, 92.5%) that COVID-19 was a dangerous disease, with a fear of being infected (21/39, 53.8%) or passing COVID-19 to friends and relatives (27/39, 69.2%). A negative correlation was observed between the students’ age and fear of getting infected with COVID-19 or infecting others, whereas older individuals viewed COVID-19 more positively. However, there were no other significant correlations between COVID-19 fears and student outcome variables.

### Limitations

Knowing the students’ perceptions allows educators to understand and adjust the teaching methodology to improve learning and engage the students using TB-learning. This analysis was limited to 1 dental school in Canada, where different strategies held during the COVID-19 pandemic might bring different outcomes. Moreover, 1 of the limitations of this work was the low response rate. Despite all the limitations encountered in a survey type of study, the use of TB-based learning in dental education and the students’ perception toward the methods and teaching techniques presented in this evaluation can give educators helpful insight into how to improve and develop an approachable and engaging web-based dental curriculum to be used after the pandemic.

### Conclusions

The fast development of TB-learning in dentistry during the COVID-19 pandemic showed differences between the web-based method and the face-to-face learning methods. There is room for improvement since the students reported fatigue with the web-based method leading to difficulty concentrating and visualizing the subject. Despite the preclinical hands-on training limitations, the students perceived having sufficient preclinical training. There was a consensus that COVID-19 affected the students’ overall learning process and practical preclinical training. Moreover, the majority of the students perceived COVID-19 as a dangerous disease and showed fear of becoming infected and infecting others.

The use of TB-learning with interactive activities is highly recommended to improve the student’s learning experience. This investigation outlines 4 variables that can lead to positive student outcomes:

- Organization of the module
- Web-based quiz questions to reinforce learning
- Relevant and quality handouts
- Good and clear audiovisuals

Focusing on improving these 4 variables will increase student satisfaction with their education. The results of this study can help guide educators in developing web-based educational modules.

## Appendix

Multimedia Appendix 1 Survey.

## Abbreviations

TB-learning: technology-based learning
WHO: World Health Organization

## Data Availability

The data sets generated during this study are available from the corresponding author on reasonable request.
