# Supplementary material for: Effects of Distance-Learning Strategies in Dental Fixed Prosthodontics Amidst the COVID-19 Pandemic: Cross-Sectional Questionnaire Study on Preclinical Dental Students’ Perspective
Source: JMIR Form Res. 2023 Nov 8;7:e45311. doi: 10.2196/45311 (PMC10666021; doi:10.2196/45311)
Supplement: Multimedia Appendix 1 [file formative_v7i1e45311_app1.docx]

| DEMOGRAPHIC QUESTIONS | | |
| --- | --- | --- |
|  | **Age** | |
|  | **Gender** | |
|  | **Year** | |
|  | **I have good internet connection quality.**  **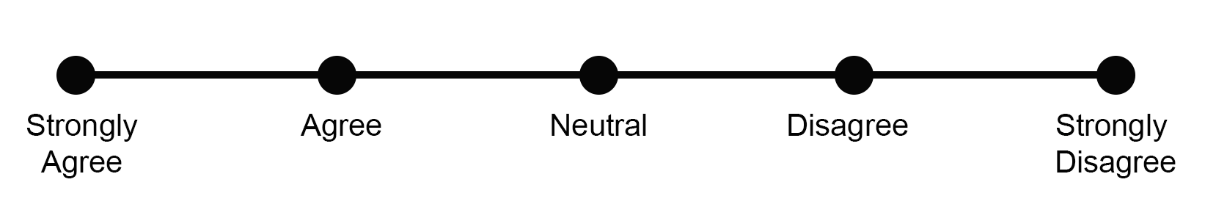** | |
|  | **I have access to Technology**  **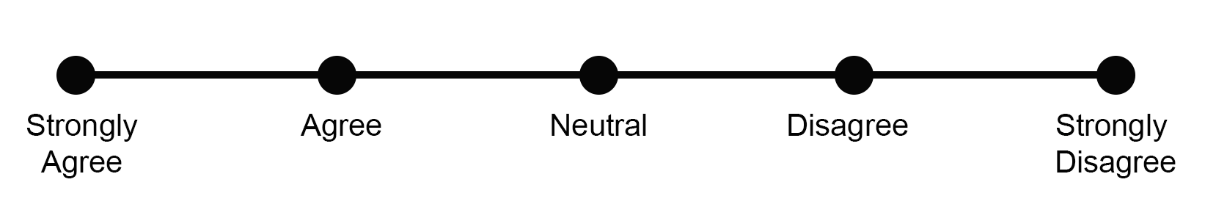** | |
|  | **Computer level skill [24]** | 5 = High level of experience  4 = Moderately high level of experience  3 = Average level of experience  2 = Low level of experience  1 = No experience |
|  | **Experience Level in (Technology-based learning) TB-learning** | 5 = High level of experience  4 = Moderately high level of experience  3 = Average level of experience  2 = Low level of experience  1 = No experience |

| TB-LEARNING |
| --- |
| 1. Technology-based learning is vital for dental education   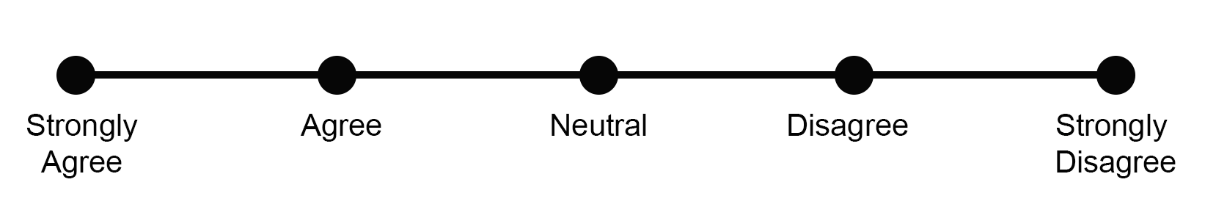 |
| 1. Technology-based learning improved my dental education   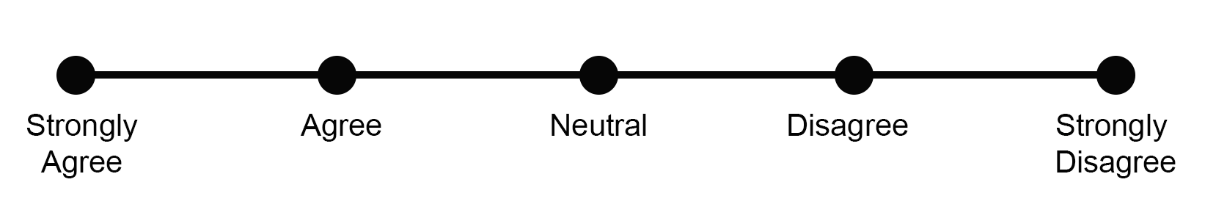 |
| 1. The information was well organized [26].   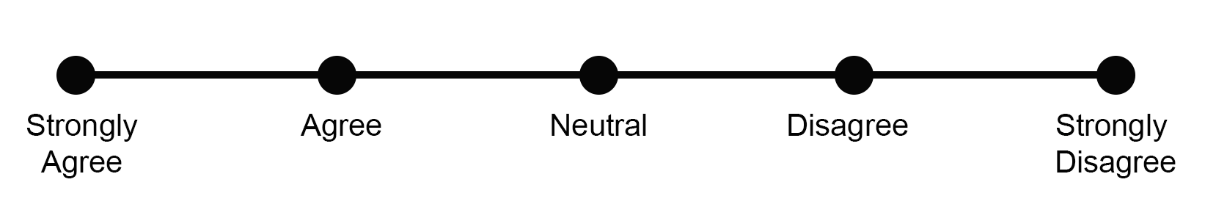 |
| 1. Using the TB-learning was fun [27]   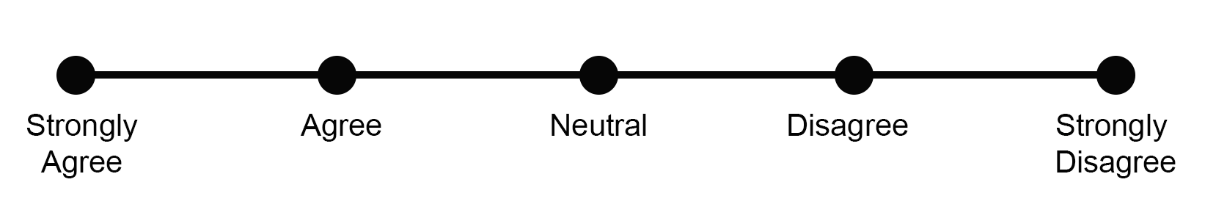 |
| 1. Using the online classes were an effective way to learn about the assigned topics   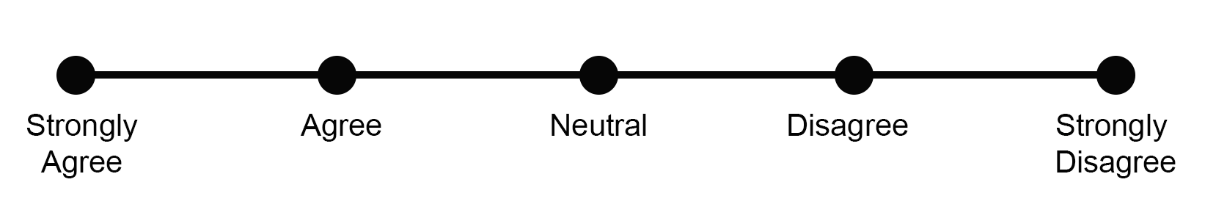 |

| 1. There was no difference between the face-to-face classes to the online classes after COVID-19   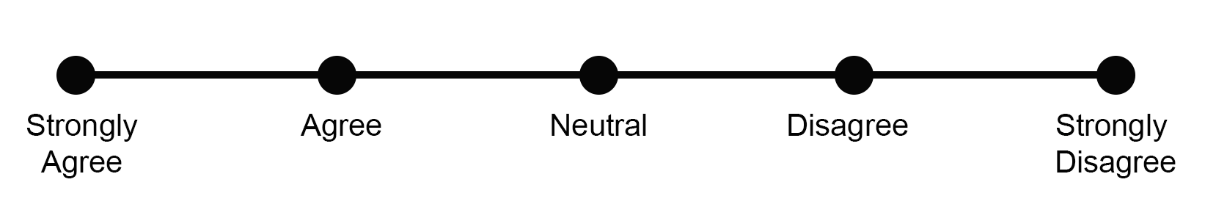 |
| --- |

| 1. **Level of engagement on my education after COVID-19**   **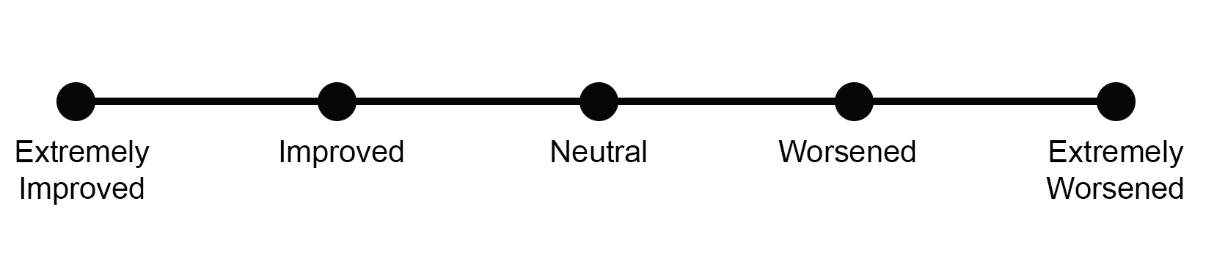** |
| --- |
| 1. **I feel fatigued from the online courses [25]**   **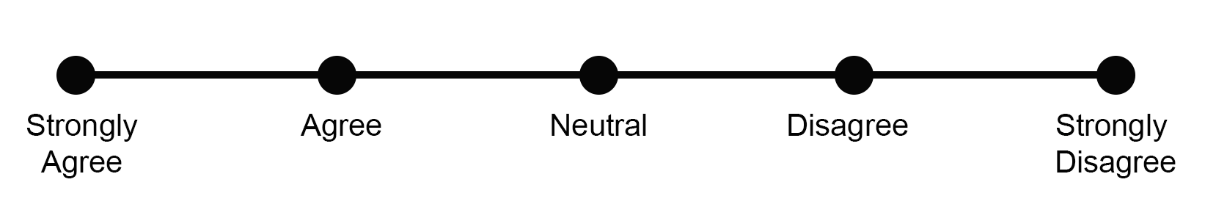** |
| 1. **I have more difficulty in concentrating in online classes compared to in-person classes? [25]**   **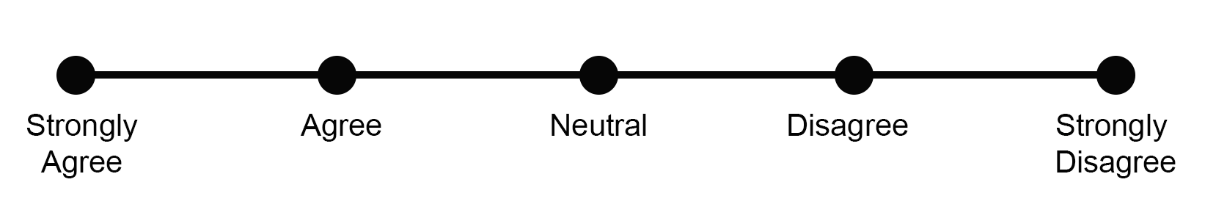** |
| 1. **I have difficulty in retaining, visualizing, or understanding the materials with the online teaching methods [25]**   **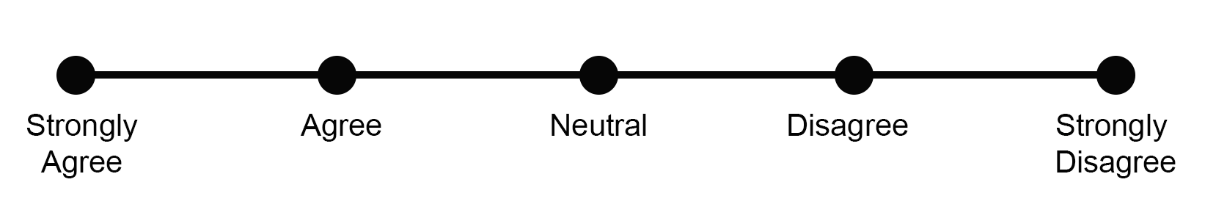** |

| 1. **The online learning approach similar to this should be used in this course in the future [27]**   **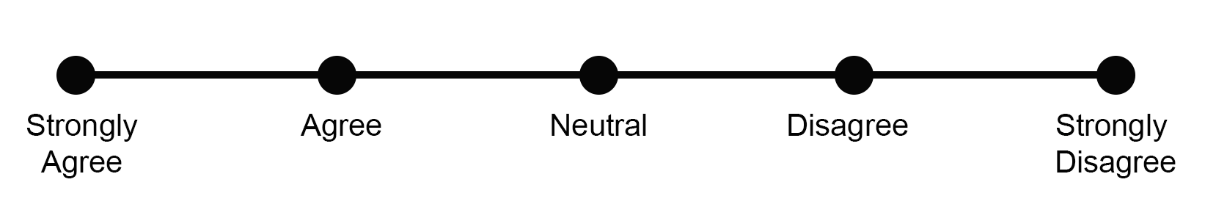** |  |
| --- | --- |
| 1. **Completing the online course did not take more time and effort compared with the face-to-face classes [27]**   **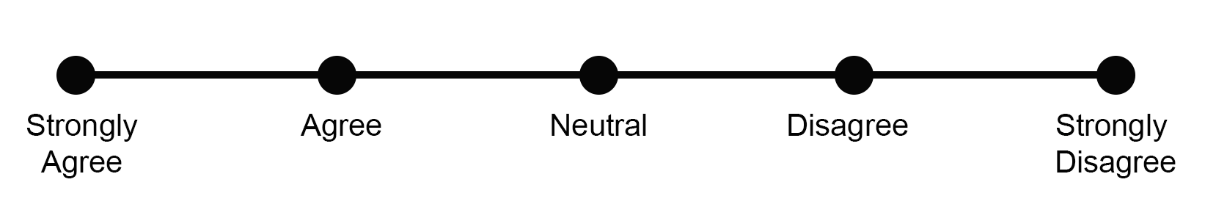** |  |
| 1. **Questions asked in the online quizzes helped me to understand the content**   **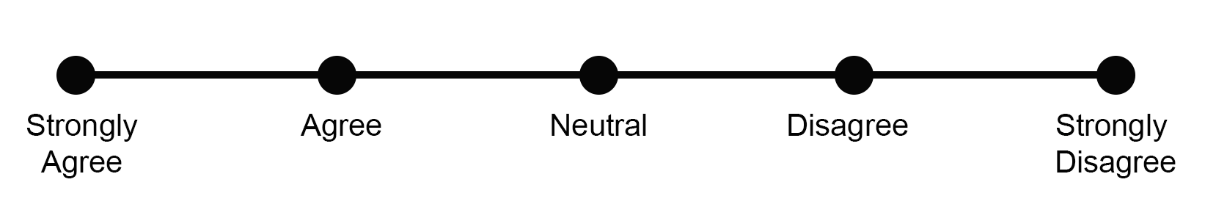** |  |
| 1. **The content of the online classes was very interesting for my preclinical training**   **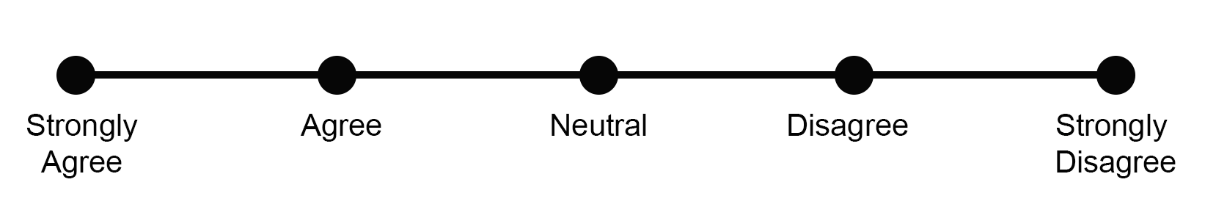** |  |
| 1. **The content of the online classes was very useful for my preclinical training**   **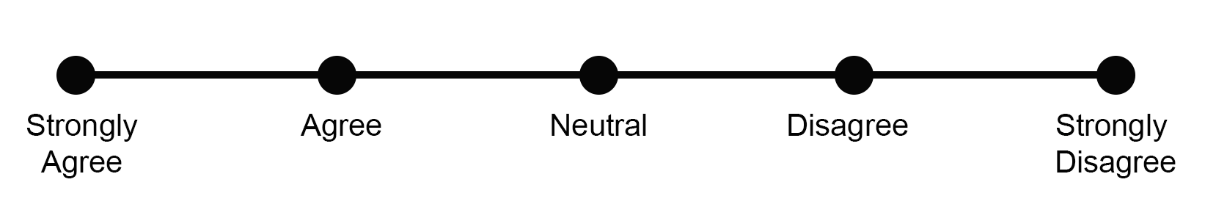** |  |
| 1. **The online content was sufficient for my preclinical training**   **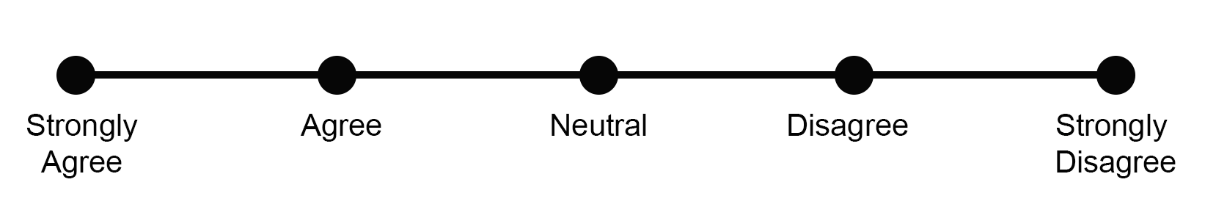** |  |
| 1. **I had no problem with the use of technology to watch the online classes**   **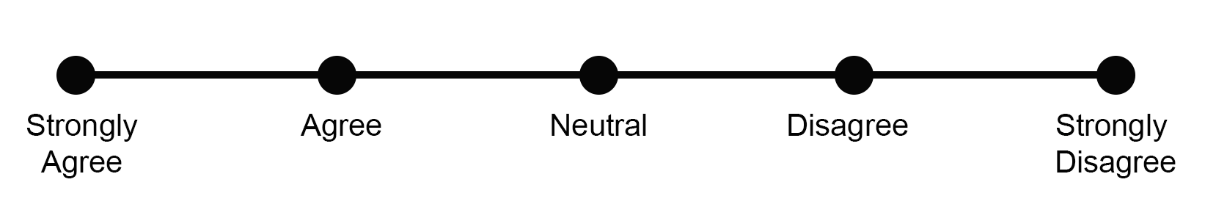** | |
| 1. **I believe that more interactive online classes will help with my education process**   **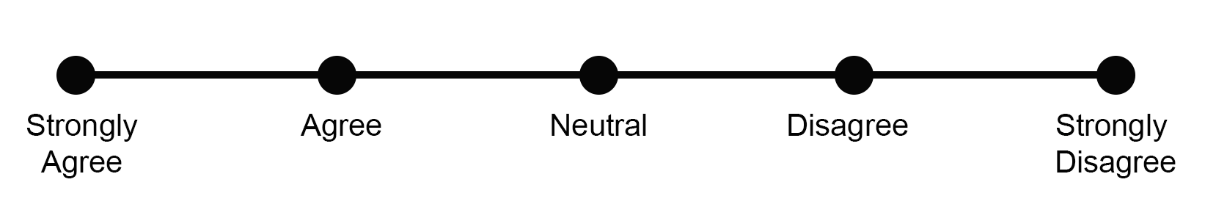** | |
| 1. **I would rather have online group discussions than watching a class**   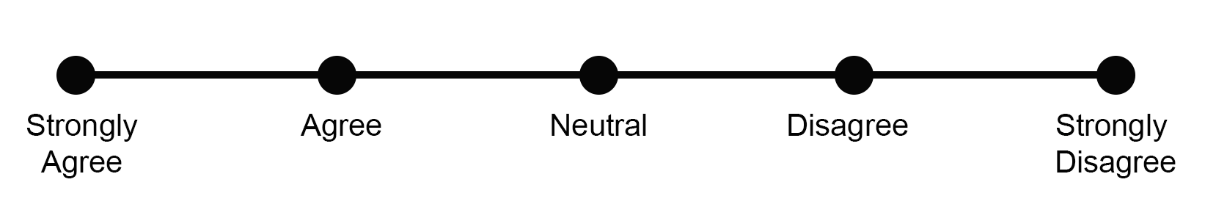 | |
| 1. **I am interested to experience Virtual Reality for my preclinical training**   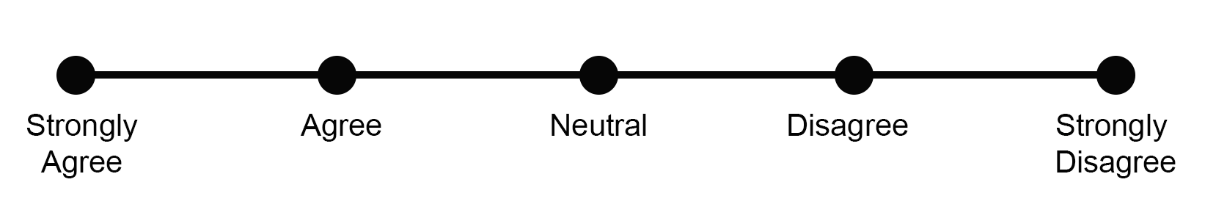 |  |

| PRECLINICAL TRAINING |
| --- |
| 1. I had sufficient hands-on preclinical training   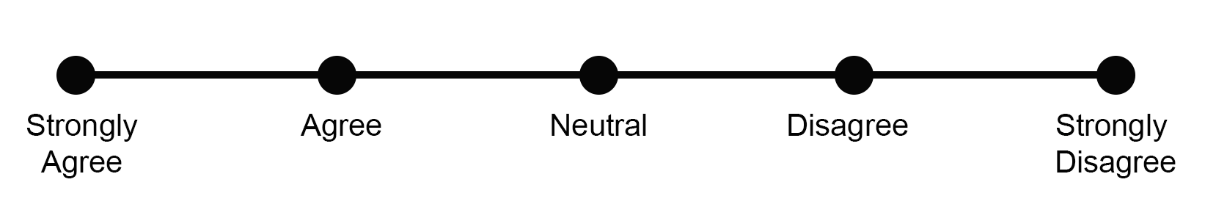 |
| 1. The audio-visuals were of high quality and it helped me with my preclinical training   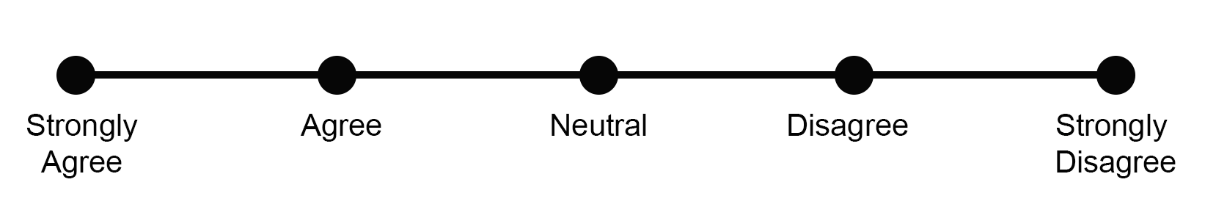 |
| 1. The information presented was applicable in my preclinical training   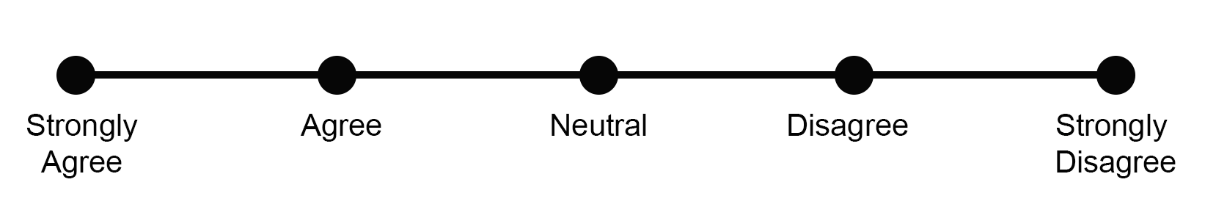 |
| 1. The handouts were relevant and helped me organize the information [26]   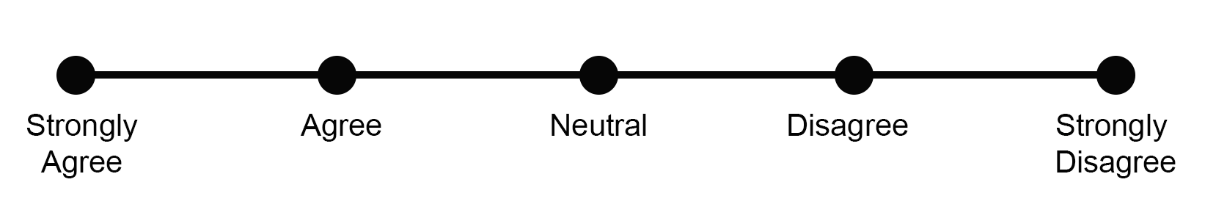 |
| 1. I had sufficient interaction with the instructor [26]   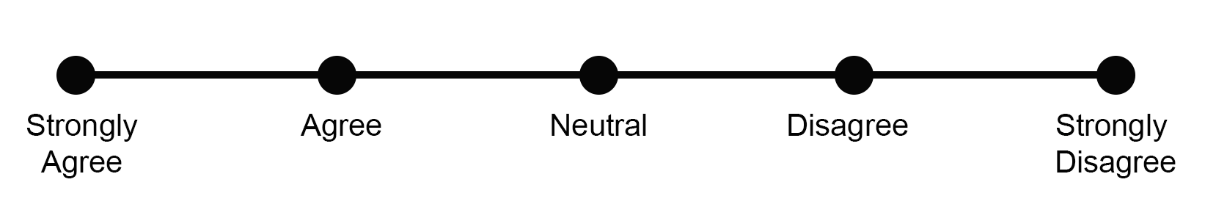 |

| 1. **I believe that videos of the preparation procedures before the laboratorial preclinical practice will help me with my preclinical training**   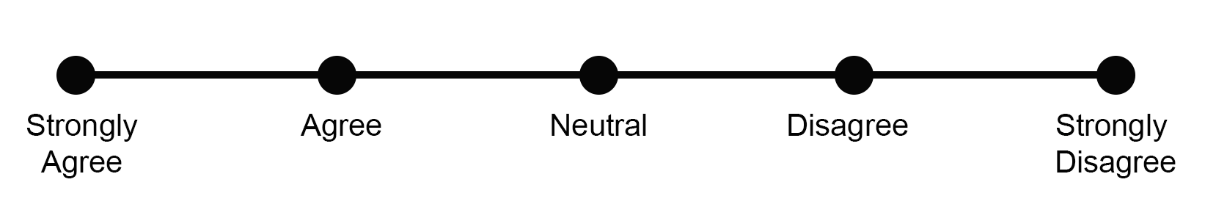 |
| --- |

| COVID-19 |
| --- |
| 1. Corona virus disease (COVID-19) is a very dangerous disease [14]   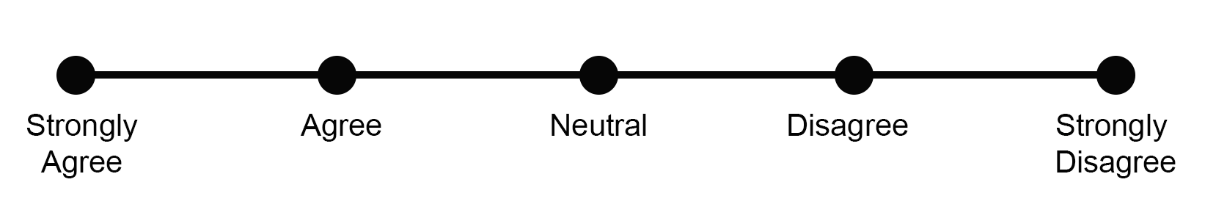 |
| 1. COVID-19 negatively affected my overall learning process in dentistry   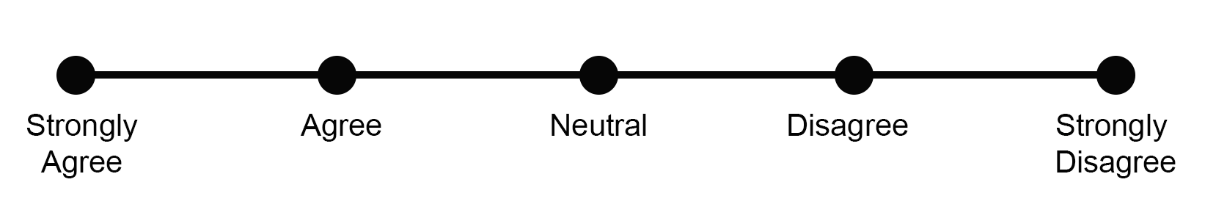 |
| 1. COVID-19 negatively affected my preclinical learning   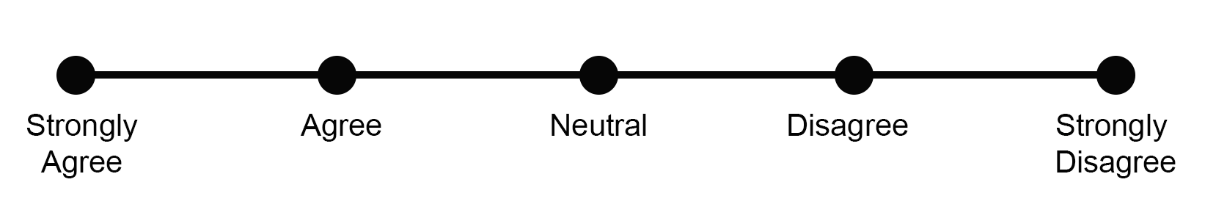 |

| 1. I am afraid of becoming infected with COVID-19 as a healthcare professional working at close range with the patient [15]   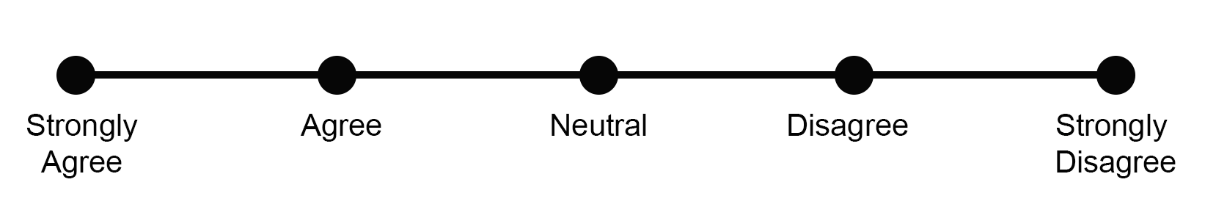 |
| --- |

| 1. I am afraid to infect any relatives or people around me in terms of COVID-19 because I am a healthcare worker working very close to the patient [15]   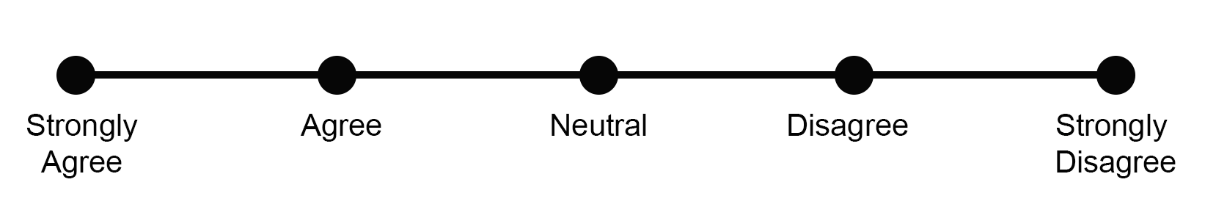 |
| --- |
| 1. With the COVID-19 outbreak, I regret that I chose the dentistry as a profession [15]   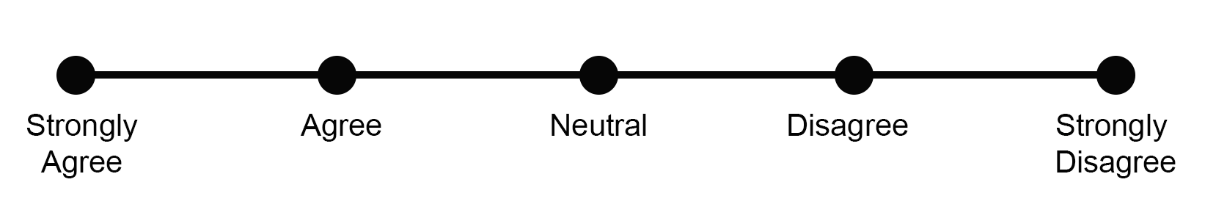 |
